# DAF-16/FOXO and HLH-30/TFEB comprise a cooperative regulatory axis controlling tubular lysosome induction in C. elegans

**DOI:** 10.21203/rs.3.rs-4049366/v1

**Published:** 2024-03-29

**Authors:** Alyssa Johnson, Cristian Ricaurte-Perez, P. Wall, Olga Dubuisson, K. Bohnert

**Affiliations:** Louisiana State University System; Louisiana State University System; Louisiana State University System; Louisiana State University System; Louisiana State University System

## Abstract

Although life expectancy has increased, longer lifespans do not always align with prolonged healthspans and, as a result, the occurrence of age-related degenerative diseases continues to increase. Thus, biomedical research has been shifting focus to strategies that enhance both lifespan and healthspan concurrently. Two major transcription factors that have been heavily studied in the context of aging and longevity are DAF-16/FOXO and HLH-30/TFEB; however, how these two factors coordinate to promote longevity is still not fully understood. In this study, we reveal a new facet of their cooperation that supports healthier aging in *C. elegans*. Namely, we demonstrate that the combinatorial effect of *daf-16* and *hlh-30* is required to trigger robust lysosomal tubulation, which contributes to systemic health benefits in late age by enhancing cross-tissue proteostasis mechanisms. Remarkably, this change in lysosomal morphology can be artificially induced via overexpression of *SVIP*, a previously characterized tubular lysosome stimulator, even when one of the key transcription factors, DAF-16, is absent. This adds to growing evidence that SVIP could be utilized to employ tubular lysosome activity in adverse conditions or disease states. Mechanistically, intestinal overexpression of *SVIP* leads to nuclear accumulation of HLH-30 in gut and non-gut tissues and triggers global gene expression changes that promotes systemic health benefits. Collectively, our work reveals a new cellular process that is under the control of DAF-16 and HLH-30 and provides further insight into how these two transcription factors may be exerting their pro-health effects.

## Introduction

The increase in life expectancy during the last century is a remarkable achievement of modern civilization. Indeed, during an interval of 73 years, from 1950 to 2023, the life expectancy at birth in the United States increased from 68.14 to 79.11 years (Nations, 2024). This has led to a growing elderly population, with the number of individuals over age 64 now exceeding the number of children under the age of five (Jaul & Barron, 2017). However, despite extended lifespans, the incidence of age-related degenerative diseases persists and may even be on the rise, indicating that individuals are not experiencing improved health in later years (Brown, 2015; Li et al, 2002). In response to the growing disparity between lifespan and healthspan, pharmacological and non-pharmacological interventions aimed at improving late age health have been tested. Among the non-pharmacological interventions, dietary restriction (DR) has been extensively studied during the last few decades, as it extends lifespan, attenuates functional decline, and delays chronic diseases across a broad variety of species (Kapahi et al, 2017; Mair & Dillin, 2008)

Although there is ample evidence supporting the beneficial impacts of DR, little is known about the cellular mechanisms underlying DR-dependent lifespan extension and healthy aging. Despite these shortcomings, the autophagy/lysosome system has been recognized as one pivotal mechanism required for the beneficial effects induced by DR; inhibiting autophagy negates the anti-aging effects of DR and abolishes lifespan extension in multiple species (Chung & Chung, 2019; Gelino et al, 2016; Hansen et al, 2008; Madeo et al, 2015). In addition to DR, several other longevity pathways converge onto autophagy (Gelino & Hansen, 2012; Hansen et al, 2018). Thus, autophagy functions as a unifying mechanism for cellular homeostasis maintenance and can facilitate cell autonomous (Gelino et al., 2016; Rana et al, 2017; Ulgherait et al, 2014) and cell nonautonomous effects (Demontis & Perrimon, 2010; Gelino et al., 2016; Ulgherait et al., 2014) to promote longevity. Similarly, proper functionality of the lysosome, the major digestive organelle that disposes and recycles autophagic cargo, is necessary to extend the lifespan of dietary restricted animals (Sun et al, 2020). Therefore, mechanisms that boost autophagy and/or lysosome function could lead to treatments that slow or reverse age-related diseases.

Lysosomes, usually depicted as spherical-shaped structures, are sophisticated organelles that play critical roles in maintaining cellular homeostasis. Although they were originally thought to be solely involved in breaking down cellular waste, it is now clear that they also play a crucial role in regulating cell metabolism and signaling (Ballabio & Bonifacino, 2020). Moreover, lysosomes exhibit a high degree of morphological plasticity; vesicular lysosomes can undergo transformation into a tubular network that facilitates processes like antigen presentation (Hipolito et al, 2019; Knapp & Swanson, 1990; Phaire-Washington et al, 1980; Saric et al, 2015; Swanson et al, 1987), cuticle remodeling (Miao et al, 2020), and autophagosome-lysosome fusion (Dolese et al, 2022; Johnson et al, 2021; Johnson et al, 2015). In previous work, our lab found that tubular lysosome (TL) formation in the gut is necessary to extend the lifespan of *C. elegans* under dietary restriction conditions (Villalobos et al, 2023). Moreover, experimentally stimulating TLs constitutively in the gut of well-fed wild-type animals is sufficient to mimic some effects of DR and promotes healthier aging (Villalobos et al., 2023). Taken together, these data suggest that TLs could represent a potential entry point for devising starvation mimetics.

Although we have gained a significant appreciation for the importance of TLs in regulating various aspects of animal physiology (Bohnert & Johnson, 2022), less is known about the molecular factors that regulate TL formation. Identifying the signaling pathways that promote the development of TLs might reveal new molecular targets to promote healthy aging. Here, we uncover a new molecular repertoire required for TL formation. We find that the transcription factors DAF-16 and HLH-30, the *C. elegans* orthologs of mammalian FOXO (Forkhead box protein O) and TFEB (Transcription Factor EB), respectively, work in concert to drive formation of gut TLs under DR and natural aging conditions. Moreover, we report that TLs can be constitutively stimulated in the gut of well-fed *daf-16* mutants by overexpressing *Drosophila* or human small VCP interacting protein (*SVIP*). Our evidence suggests that SVIP bypasses the requirement for DAF-16 by triggering more robust HLH-30 activation to induce TL formation. Remarkably, precocious TL induction in the gut reduces cellular hallmarks of aging and promotes late-age health in short-lived *daf-16* mutants, underscoring the anti-aging properties of TLs. Mechanistically, *SVIP* overexpression in the gut stimulates HLH-30 activation across multiple tissues, triggering global gene expression changes that facilitate systemic health improvements. Collectively, our results suggest that a DAF-16 and HLH-30 regulatory axis controls TL formation under different conditions and further underscore SVIP as a potential interventional target for anti-aging therapies.

## Results

### DAF-16/FOXO and HLH-30/TFEB are each required for robust TL induction in the *C. elegans* gut

**Figure 1.**
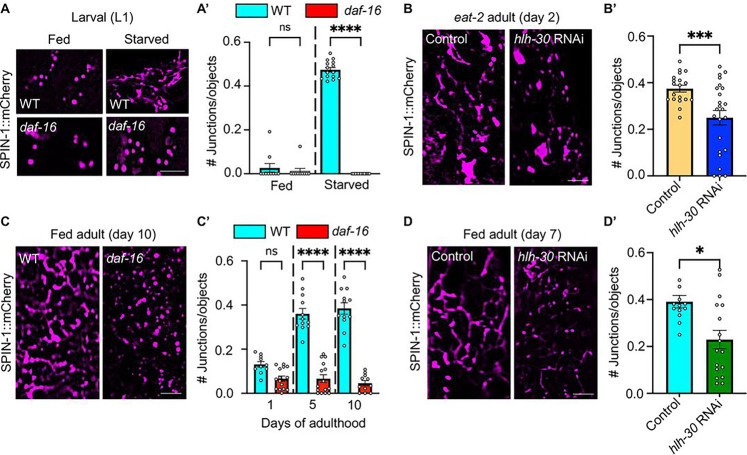
DAF-16/FOXO and HLH-30/TFEB are each required for robust TL induction in the *C. elegans* gut. **(A)** Endogenously tagged SPIN-1::mCh in fed and starved wild-type and *daf-16* worms at L1 larval stage. Scale bar, 5 μm. **(A’)** Endogenously tagged SPIN-1::mCh junctions/object in fed and starved wild-type and *daf-16* worms at L1 larval stage (*n*=10 well-fed worms for each genotype and *n*=15 starved worms for each genotype). Mean ± s.e.m. Two-way ANOVA with Šídák’s multiple comparisons. (ns=not significant, ****p<0.0001). **(B)** Endogenously tagged SPIN-1::mCh in day 2 *eat-2* animals that were fed control or *hlh-30* RNAi beginning at L4 larval stage. Scale bar, 5 μm. **(B’)** Endogenously tagged SPIN-1::mCh junctions/object in day 2 *eat-2* animals that were fed control or *hlh-30* RNAi beginning at L4 larval stage. (*n*=18 fed control worms and *n*=23 fed *hlh-30* RNAi worms). Mean ± s.e.m. Unpaired two-tailed Student’s t-test. (***p<0.001). **(C)** Endogenously tagged SPIN-1::mCh in fed wild-type and *daf-16* worms at day 10 of adulthood. Scale bar, 5 μm. **(C’)** Endogenously tagged SPIN-1::mCh junctions/object in fed wild-type and *daf-16* worms at days 1, 5 and 10 of adulthood. (*n*=10 wild-type worms day 1, *n*=12 wild-type worms day 5, *n*=12 wild-type worms day 10, *n*=15 *daf-16* worms day 1, *n*=15 *daf-16* worms day 5, and *n*=11 *daf-16* worms day 10). Mean ± s.e.m. Two-way ANOVA with Tukey’s multiple comparisons. (ns=not significant, ****p<0.0001). **(D)** Endogenously tagged SPIN-1::mCh in day 7 wild-type animals that were fed control or *hlh-30* RNAi at day 5 of adulthood. Scale bar, 5 μm. **(D’)** Endogenously tagged SPIN-1::mCh in day 7 wild-type animals that were fed control or *hlh-30* RNAi at day 5 of adulthood. (*n*=13 fed control worms and *n*=17 fed *hlh-30* RNAi worms). Mean ± s.e.m. Unpaired two-tailed Student’s t-test. (*p<0.05).

Although lysosomes have been canonically described as spherical-shaped organelles (Bainton, 1981), we and others have shown that vesicular lysosomes can morph into degradative tubular networks under certain stimuli (Dolese et al., 2022; Hipolito et al, 2018; Johnson et al., 2015; Ramos et al, 2022; Saric et al., 2015; Swanson et al., 1987; Villalobos et al., 2023). Notably, we found that autophagic TLs are robustly stimulated in the gut of dietary restricted *C. elegans* and are required to elicit the full beneficial effects of DR (Villalobos et al., 2023). However, there is limited understanding of the molecular repertoire necessary to coordinate the formation of TLs, and it remains unknown whether TLs may also contribute to other longevity paradigms beyond DR. Thus, to identify new molecular factors required for initiating TL formation, we performed a candidate-based screen in starved or dietary restricted *C. elegans* using genetic mutations or RNAi-based inhibition. Because TL stimulation in the gut provides pro-health effects, we focused on genes that have been established to affect longevity (Extended data, Fig. 1). To visualize lysosomes in their most natural context, we imaged a previously characterized fluorescent marker that has an mCherry tag incorporated at the endogenous C-terminus of the lysosomal membrane protein Spinster/SPIN-1 (SPIN-1::mCherry) (Ramos et al., 2022; Villalobos et al., 2023) .

While several mutations, including *jnk-1,aak-2, clk-1,* and *pdk-1*, had little effect on starvation-induced TLs (Extended data, Fig. 1), our screen revealed multiple genes that when mutated or inhibited blocked the formation of TLs during starvation or DR. Notably, starved mutants lacking DAF-16/FOXO, a key pro-longevity transcription factor in the insulin/IGF-1 signaling pathway (Kenyon et al, 1993; Sun et al, 2017), were unable to deploy TLs (Fig. 1A–A’, Extended data, Fig. 1). Likewise, we found that HLH-30/TFEB, a transcription factor that acts as a master regulator of lysosomal biogenesis and promotes health and longevity (Lapierre et al, 2013), was also required for TL induction during DR. Specifically, RNAi knockdown of *hlh-30* in *eat-2* mutants, a genetic model for DR (Lakowski & Hekimi, 1998), impeded TL formation (Fig. 1B–B’). Thus, *daf-16* and *hlh-30* are each required for starvation-dependent TL induction.

Previously, we found that TLs are also naturally stimulated in late-age *C. elegans* (Villalobos et al., 2023); thus, we further explored whether *daf-16* and *hlh-30* are required for TL formation during natural aging. To assess the requirement of *daf-16*, we imaged lysosomes in well-fed *daf-16* mutants at days 1, 5, and 10 of adulthood and found that *daf-16* mutants were unable to efficiently induce TLs in late adulthood compared to wild-type counterparts (Fig. 1C–C’). To assess the dependency on *hlh-30*, we treated worms at day 5 of adulthood with control or *hlh-30* RNAi and assessed TL integrity two days later (i.e., on day 7 of adulthood). Similar to *daf-16* mutants, *hlh-30*-RNAi animals also showed reduced TL formation in mid-to late-age (Fig. 1D–D’). These data indicate that DAF-16/FOXO and HLH-30/TFEB, two major transcription factors that regulate organismal longevity, are required to induce robust TL formation in biological contexts in which there is a high autophagic demand, such as nutrient deprivation and aging.

### Overexpression of either *daf-16* or *hlh-30* is sufficient to induce lysosomal tubulation in young well-fed animals.

**Figure 2.**
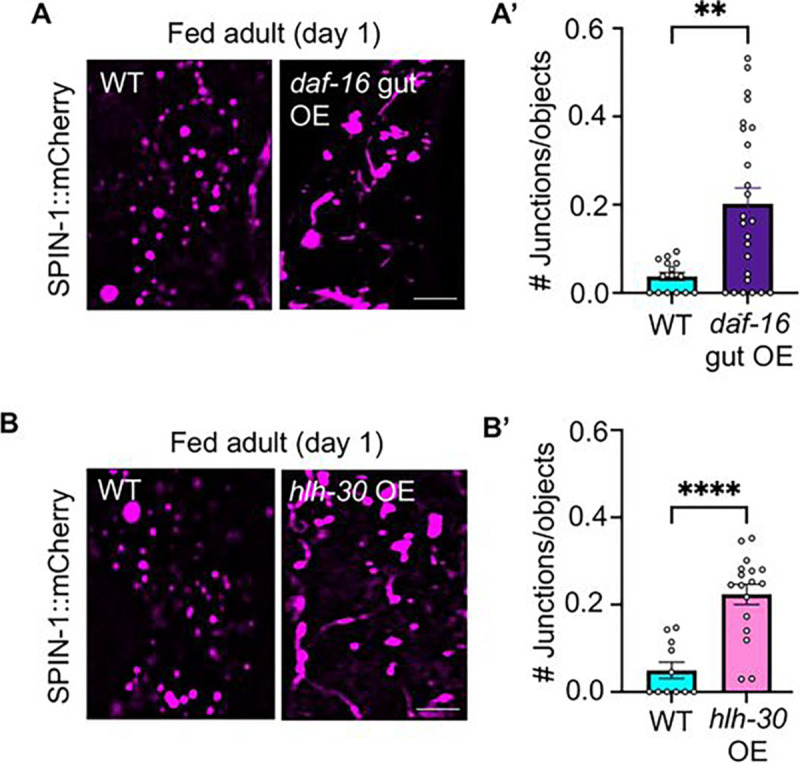
Overexpression of either *daf-16* or *hlh-30* is sufficient to induce lysosomal tubulation in young well-fed animals. **(A)** Endogenously tagged SPIN-1::mCh in fed wild-type and gut *daf-16* OE animals at day 1 of adulthood. Scale bar, 5 μm. **(A’)** Endogenously tagged SPIN-1::mCh junctions/object in fed wild-type and gut *daf-16* OE animals at day 1 of adulthood. (*n*=15 wild-type worms and *n*=25 *daf-16* OE worms). Mean ± s.e.m. Unpaired two-tailed Student’s t-test. (**p<0.01). **(B**) Endogenously tagged SPIN-1::mCh in fed wild-type and *hlh-30* OE animals at day 1 of adulthood. Scale bar, 5 μm. **(B’)** Endogenously tagged SPIN-1::mCh junctions/object in fed wild-type and *hlh-30* OE animals at day 1 of adulthood. (*n*=11 wild-type worms and *n*=17 *hlh-30* OE worms). Mean ± s.e.m. Unpaired two-tailed Student’s t-test. (****p<0.0001).

Given that overexpression of either *daf-16* or *hlh-30* promotes longevity (Lapierre et al., 2013; Lin et al, 2001; Settembre et al, 2011), we next explored whether overexpression of either transcription factor would be sufficient to drive the morphological transition of lysosomes from vesicles to tubules. If so, this could indicate that TL induction possibly contributes to the longevity effects of *daf-16* and/or *hlh-30*. To examine this possibility, we first overexpressed *daf-16* in the gut using the gut-specific promoter, Pges-1 (Kennedy et al, 1993), and visualized lysosomes using the endogenous SPIN-1::mCherry marker. Indeed, we found that intestinal overexpression of *daf-16* was sufficient to induce TLs in the gut of young (day 1) well-fed adults (Fig. 2A–A’), which normally do not show TLs (Fig. 2A–B, (Villalobos et al., 2023)). We next examined the effect of *hlh-30* overexpression. *hlh-30* was expressed in several copies per cell from the extrachromosomal array *Phlh-30*::*hlh-30*::GFP (Lapierre et al., 2013). Similar to *daf-16* overexpression, *hlh-30* overexpression also triggered TL formation in young well-fed adults (Fig. 2B–B’). Notably, these data are consistent with our previous findings that inhibition of mTOR signaling, a known trigger for HLH-30 activation (Roczniak-Ferguson et al, 2012), also stimulates TL formation in young well-fed animals (Villalobos et al., 2023). Collectively, these results indicate that experimental overexpression of either *hlh-30* or *daf-16* is sufficient to stimulate TL induction in the *C. elegans* gut.

### Gut-specific activity of DAF-16/FOXO in lifespan extension and healthy aging depends on TL formation.

**Figure 3.**
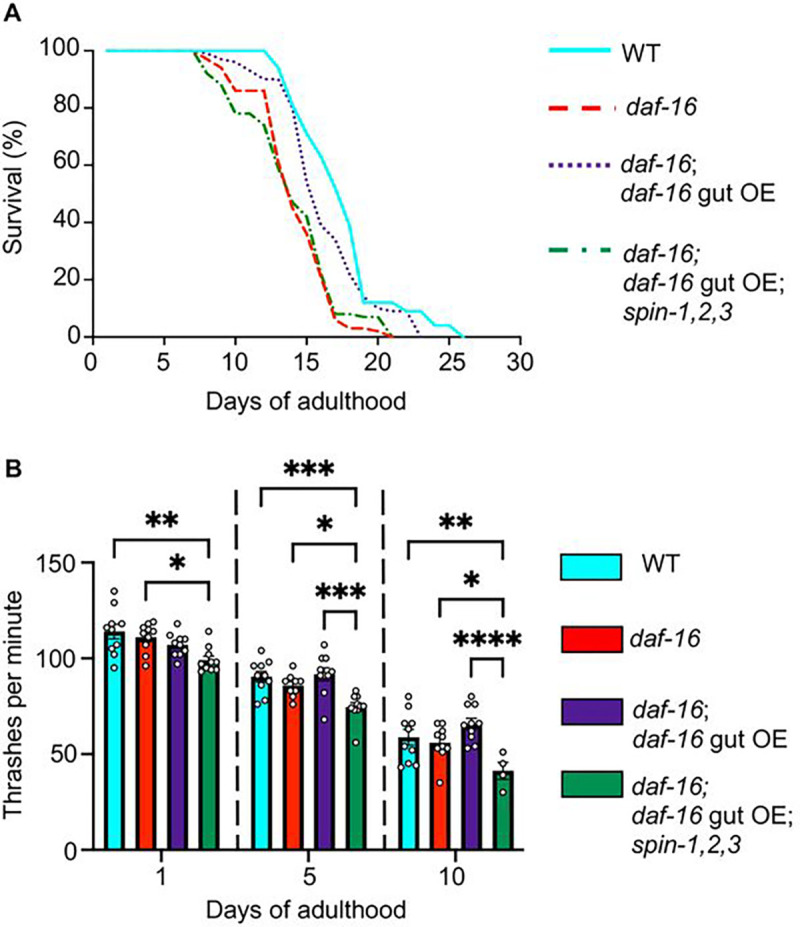
Gut-specific activity of DAF-16/FOXO in lifespan extension and healthy aging depends on TL formation. **(A)** Lifespan of the indicated genotypes. A log-rank test was used to determine statistical significance. For statistics, see Supplementary Table 2. **(B)** Thrashing rates of the genotypes indicated. (*n*=10 worms per condition). Mean ± s.e.m. Two-way ANOVA with Šídák’s multiple comparisons. (*p<0.05, **p<0.01, ***p<0.001, ****p<0.0001)

Mutations in the transcription factor DAF-16 shorten the lifespan of wild-type *C. elegans* (Kenyon et al., 1993). However, restoring *daf-16* expression specifically in the gut, rather than in other tissues, can restore lifespan back to near wild-type (Libina et al, 2003). Thus, *daf-16* gut-specific activity plays a key role in regulating longevity. Given that *daf-16* overexpression is sufficient to trigger TL induction and that TL induction in the gut alone can promote healthier aging (Villalobos et al., 2023), we next explored whether TLs are required for *daf-16* gut-specific activity in longevity. In previous work, we demonstrated that TLs can be genetically blocked by simultaneous mutation of three *spin* paralogs (*spin-1,2,3* triple mutant) (Villalobos et al., 2023). Thus, we used this strategy to prevent TL formation in *daf-16* mutant animals that also overexpressed *daf-16* exclusively in the gut (*spin-1,2,3; daf-16(mu86); Pges-1::daf-16*). While re-expression of *daf-16* in the gut of *daf-16* mutants increased lifespan back to near wild-type levels as previously reported (Libina et al., 2003), we observed no significant extension of lifespan when TL formation was genetically blocked (Fig. 3A). These data suggest that TL activity is required for the gut-specific effect of *daf-16* on longevity.

We next examined if preventing TLs would also abrogate aspects of late-age health improvements seen upon *daf-16* re-expression in the gut of *daf-16* mutants (Libina et al., 2003). While *daf-16* null mutants with *daf-16* re-expression in the gut demonstrated improved late-age mobility compared to *daf-16* mutants alone, we found no significant improvement to late-age mobility when TL formation was impeded in this context (Fig. 3B). These data suggest that TL formation is a necessary step for the gut-specific actions of DAF-16 in promoting organismal health and longevity and highlight that TLs contribute to longevity paradigms beyond DR.

### Forced tubular lysosome induction promotes healthy aging in *daf-16* mutants.

**Figure 4.**
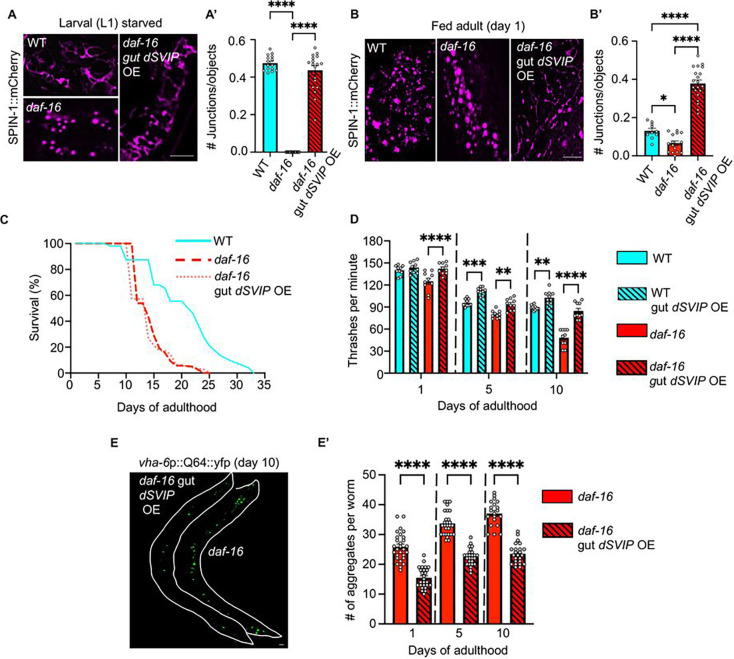
Forced tubular lysosome induction promotes healthy aging in *daf-16* mutants. **(A)** Endogenously tagged SPIN-1::mCh in starved wild-type, *daf-16*, and *daf-16* gut *dSVIP* OE animals at L1 larval stage. Scale bar, 5 μm. **(A’)** Endogenously tagged SPIN-1::mCh junctions/object in starved wild-type, *daf-16*, and *daf-16* gut *dSVIP* OE animals at L1 larval stage. (*n*=15 wild-type worms, *n*=15 *daf-16* worms, and *n*=19 *daf-16* gut *dSVIP* OE worms). Mean ± s.e.m. One-way ANOVA with Tukey’s multiple comparisons. (****p<0.0001). **(B)** Endogenously tagged SPIN-1::mCh in fed wild-type, *daf-16*, and *daf-16* gut *dSVIP* OE animals at day 1 of adulthood. Scale bar, 5 μm. **(B’)** Endogenously tagged SPIN-1::mCh junctions/object in fed wild-type, *daf-16*, and *daf-16* gut *dSVIP* OE animals at day 1 of adulthood. (*n*=10 wild-type worms, *n*=15 *daf-16* worms, and *n*=20 *daf-16* gut *dSVIP* OE worms). Mean ± s.e.m. One-way ANOVA with Tukey’s multiple comparisons. ( ****p<0.0001). **(C)** Lifespan of the indicated genotypes. A log-rank test was used to determine statistical significance. For statistics, see Supplementary Table 2. **(D)** Thrashing rates of the genotypes indicated. (*n*=10 worms per condition). Mean ± s.e.m. Two-way ANOVA with Šidák’s multiple comparisons. (**p<0.01, ***p<0.001, ****p<0.0001). **(E)** Gut-expressed Q64::YFP in *daf-16* and *daf-16* gut *dSVIP* OE at day 10 of adulthood. Scale bar, 100 μm. **(E’)** Number of gut Q64::YFP aggregates in *daf-16* and *daf-16* gut *dSVIP* OE at day 10 of adulthood (*n*=28 *daf-16* worms day 1, *n*=28 *daf-16* worms day 5, *n*=20 *daf-16* worms day 10, *n*=26 *daf-16* gut *dSVIP* OE worms day 1, *n*=26 *daf-16* gut *dSVIP* OE worms day 5, and *n*=24 *daf-16* gut *dSVIP* OE worms day 10). Mean ± s.e.m. One-way ANOVA with Šidák’s multiple comparisons (****p<0.0001).

Because we found that *daf-16* mutants are unable to form TLs (Fig. 1A–A’ and Fig. 1C–C’) and that TLs are required for some aspects of *daf-16*-dependent longevity (Fig. 3A), we were curious if forcing TL induction could overcome the *daf-16*-dependent constraints on longevity. In a prior study, we reported that overexpression of *Drosophila SVIP (dSVIP)*, a previously characterized TL stimulator (Johnson et al., 2021), induces TLs constitutively when expressed in the *C. elegans* gut, even under well-fed conditions (Villalobos et al., 2023). Thus, we tested whether overexpression of *dSVIP* in the gut of *daf-16* mutants could forcibly induce TL stimulation. Remarkably, *daf-16* mutants with gut *dSVIP* overexpression formed TLs under both fed and starved conditions (Fig. 4A–A’, B–B’). These data indicate that overexpression of *dSVIP* can bypass the genetic requirement for *daf-16* to trigger TLs.

Previously, we also reported that *SVIP*-dependent TL induction requires the activity of VCP, a AAA+ ATPase that is recruited to lysosomes upon *SVIP* overexpression and promotes lysosomal membrane fusion (Johnson et al., 2021; Villalobos et al., 2023). Thus, we examined whether SVIP-dependent TL induction in *daf-16* mutants was also VCP-dependent. Indeed, feeding worms a chemical inhibitor of VCP activity (CB5083) precluded TL induction in *daf-16* mutants overexpressing *SVIP* (Extended data, Fig. 2A-A’). Unexpectedly, we found that TLs triggered by natural aging or nutrient deprivation were not impeded by VCP inhibition (Extended data, Fig. 2B-B’ and C-C’). These data indicate that VCP is required for SVIP-specific TL induction, including in the absence of *daf-16*, but it is not a required factor for all modes of TL stimulation. Thus, TLs can be stimulated via multiple mechanisms.

We next examined the physiological ramifications of forced TL induction in *daf-16* mutants. We first tested whether artificial TL induction via *dSVIP* gut overexpression could extend the lifespan of short-lived *daf-16* mutants. Despite having strong TL induction, *daf-16* mutants overexpressing *dSVIP* in the gut did not show an improved lifespan relative to *daf-16* controls (Fig. 4C). This is consistent with our prior observations that *dSVIP* gut overexpression in wild-type *C. elegans* likewise does not extend lifespan (Villalobos et al., 2023). However, in our previous work, we found that while there was no effect on lifespan, late-age mobility was significantly improved in animals overexpressing *dSVIP* in the gut. Thus, we wondered whether, despite having no effect on lifespan, *dSVIP* gut overexpression could improve the healthspan of *daf-16* mutants. To evaluate this, we assayed mobility decline throughout life, a strong correlate of healthspan (Hahm et al, 2015), in *daf-16* mutants as well as in *daf-16* mutants with *dSVIP* gut overexpression. Remarkably, *daf-16* mutant animals overexpressing *dSVIP* in the gut demonstrated improved late-age mobility compared to *daf-16* mutants without *dSVIP* overexpression (Fig. 4D).

To further assess the pro-health effects of *dSVIP* gut overexpression in *daf-16* mutants, we examined effects on proteostasis, since proteostasis collapse is a major hallmark of aging (David et al, 2010b). We overexpressed self-aggregating fluorescent polyglutamine proteins (polyQ) in the gut, which accelerates cellular and organismal aging phenotypes (David et al, 2010a; Morley et al, 2002). Strikingly, the number of Q64 aggregates was notably reduced throughout the life of *daf-16* mutants overexpressing *dSVIP* in the gut (Fig. 4E–E’). Taken together, our data support the notion that TL induction driven by gut *dSVIP* overexpression promotes healthier aging in short-lived *daf-16* mutants, as in wild-type animals (Villalobos et al., 2023), and lends further support to our model that SVIP-dependent TL induction specifically improves healthspan without affecting lifespan.

### *SVIP* overexpression induces HLH-30 translocation in multiple tissues independently of DAF-16

**Figure 5.**
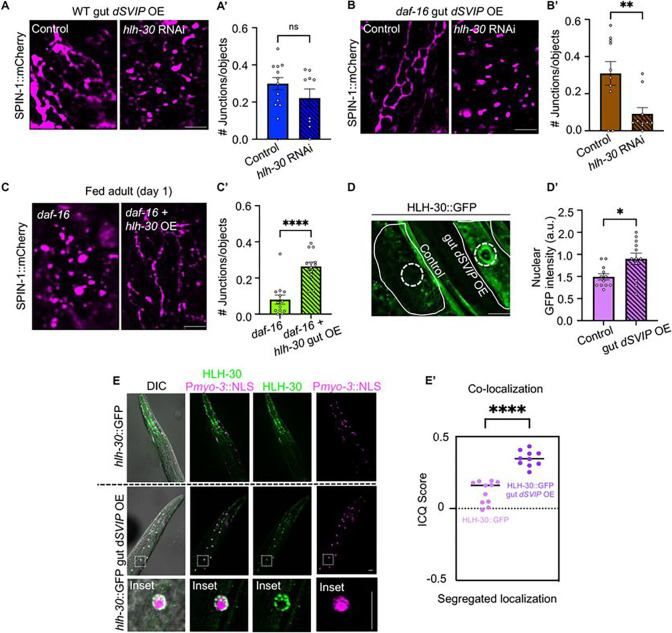
*SVIP* overexpression induces HLH-30 translocation in multiple tissues independently of DAF-16. **(A)** Endogenously tagged SPIN-1::mCh in wild-type gut *dSVIP* OE animals at day 1 of adulthood that were fed control or *hlh-30* RNAi beginning at L4 larval stage. Scale bar, 5 μm. **(A’)** Endogenously tagged SPIN-1::mCh junctions/object in wild-type gut *dSVIP* OE animals at day 1 of adulthood that were fed control or *hlh-30* RNAi beginning at L4 larval stage. (*n*=12 fed control worms and *n*=9 fed *hlh-30* RNAi worms). Mean ± s.e.m. Unpaired two-tailed Student’s t-test. (ns=not significant). **(B)** Endogenously tagged SPIN-1::mCh in *daf-16* gut *dSVIP* OE animals at day 1 of adulthood that were fed control or *hlh-30* RNAi beginning at L4 larval stage. Scale bar, 5 μm. **(B’)** Endogenously tagged SPIN-1::mCh junctions/objects in *daf-16* gut *dSVIP* OE animals at day 1 of adulthood that were fed control or *hlh-30* RNAi beginning at L4 larval stage. (*n*=10 worms per condition). Mean ± s.e.m. Unpaired two-tailed Student’s t-test (**p<0.01). **(C)** Endogenously tagged SPIN-1::mCh in fed *daf-16* mutants and *daf-16* mutants overexpressing *hlh-30* at day 1 of adulthood. Scale bar, 5 μm. **(C’)** Endogenously tagged SPIN-1::mCh junctions/object in fed *daf-16* mutants and *daf-16* mutants overexpressing *hlh-30* animals at day 1 of adulthood. (*n*=12 worms per condition). Mean ± s.e.m. Unpaired two-tailed Student’s t-test. (****p<0.0001). **(D)** HLH-30::GFP localization in gut cells of wild-type animals with and without gut *dSVIP* OE. Solid line outlines an individual gut cell and dotted line indicates nucleus. Scale bar, 5 μm. **(D’)** Quantification of HLH-30::GFP nuclear localization in gut cells of wild-type animals with and without gut *dSVIP* OE (*n*=12 worms per condition). Mean ± s.e.m. Unpaired two-tailed Student’s t-test (*p<0.05). **(E)** Colocalization of HLH-30::GFP and P*myo-3*::NLS::mCh in wild-type animals with and without gut dSVIP OE. First panel is merged DIC (Differential interference contrast) image, GFP (HLH-30) and mCherry (P*myo*::NLS). Second panel is overlay of GFP (HLH-30) and mCherry (P*myo*::NLS). Third and fourth panels are single fluorescent channels. Enlarged insets are shown to demonstrate GFP (HLH-30) and mCherry (P*myo*::NLS) colocalization. Scale bar, 5 μm. **(E’)** ICQ score were quantified in whole body of wild-type animals with and without gut *SVIP* OE. Intensity correlation quotient (ICQ) values between 0 and 0.5 indicate colocalization and values between 0 and −0.5 indicate segregated fluorescence. (*n*=11 HLH-30::GFP worms and *n*=10 HLH-30::GFP gut *dSVIP* OE worms). Mean ± s.e.m. Unpaired two-tailed Student’s t-test (****p<0.0001).

We next explored how SVIP bypasses the requirement of *daf-16* in TL induction. Given that overexpression of either *daf-16* or *hlh-30* alone is sufficient to stimulate TLs constitutively in well-fed animals, we surmised that perhaps SVIP is acting on HLH-30 as an alternative mechanism to induce TLs in the absence of *daf-16*. To test this, we knocked down *hlh-30* via RNAi in wild-type and *daf-16* animals overexpressing *dSVIP* in the gut. While TL formation was modestly reduced by *hlh-30* RNAi in wild-type *C. elegans* overexpressing *dSVIP* in the gut (Fig. 5A–A’), knockdown of *hlh-30* in *daf-16* mutants with *dSVIP* overexpression nearly abolished lysosomal tubulation (Fig. 5B–B’). This suggests that SVIP likely acts on either transcription factor to induce TLs but if one transcription factor is absent, the other can partially compensate. If this is the case, one would then expect that overexpression of HLH-30 on its own would also induce TLs in the absence of *daf-16*. To test this, we overexpressed *hlh-30* in *daf-16* mutants and examined lysosome morphology. Indeed, we found that overexpression of *hlh-30* in *daf-16* mutants triggered TLs constitutively (Fig. 5C–C’). Taken together, these data underscore the cooperative activity of DAF-16 and HLH-30 in triggering TL formation and indicate that SVIP relies more heavily on HLH-30 in the absence of *daf-16*.

HLH-30 activity is predominantly regulated by its subcellular localization; under basal conditions, HLH-30 resides in the cytoplasm but when stimulated translocates into the nucleus where it activates target genes (Roczniak-Ferguson et al., 2012). To further determine whether SVIP induces HLH-30 activation, we analyzed nuclear accumulation of HLH-30::GFP in the presence and absence of intestinal *dSVIP* overexpression. Consistent with the idea that SVIP activates HLH-30, we observed strong HLH-30 accumulation in the nucleus of gut cells in strains overexpressing *dSVIP* in the gut (Fig. 5D–D’). Moreover, in our observations, we also noticed potential HLH-30 nuclear localization in non-gut tissues. In particular, we observed potential HLH-30 nuclear localization in muscle tissues. Thus, we investigated this possibility further by co-imaging a muscle-specific nuclear marker (*Pmyo-3::NLS:mCherry*) with HLH-30::GFP in control worms and in worms overexpressing *dSVIP* in the gut. Remarkably, we observed strong co-localization of GFP and mCherry signals only in strains overexpressing gut *dSVIP* (Fig. 5E–E’). This indicates that *dSVIP* overexpressed in the gut activates HLH-30 in distinct tissues, most notably muscle. Moreover, these data suggest that *dSVIP* overexpression in the gut might elicit systemic benefits via cross tissue HLH-30 activation.

### Overexpression of *dSVIP* in the intestine reprograms the transcriptome in wild-type and *daf-16* mutants

**Figure 6.**
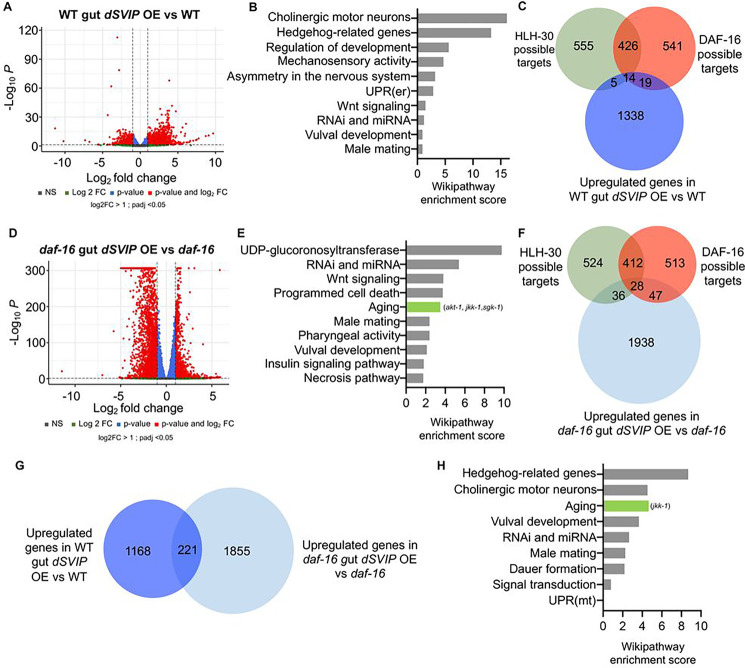
Overexpression of *dSVIP* in the intestine reprograms the transcriptome in wild-type and *daf-16* mutants. **(A)** Volcano plots illustrating gene expression changes when *dSVIP* is overexpressed in the gut of wild-type worms vs. wildtype strains. **(B)** Wikipathway enrichment analysis of the upregulated genes in wild-type strains overexpressing *dSVIP* in the gut vs. wild-type. **(C)** Venn diagram illustrating overlap between upregulated genes in wild-type animals overexpressing *dSVIP* in the gut vs. wild-type and possible targets of HLH-30 and DAF-16. **(D)** Volcano plots illustrating gene expression changes when *dSVIP* is overexpressed in the gut of *daf-16* mutants vs. *daf-16* mutants. **(E)** Wikipathway enrichment analysis of the upregulated genes in *daf-16* mutants overexpressing *dSVIP* in the gut vs. *daf-16* mutants. **(F)** Venn diagram illustrating overlap between upregulated genes in *daf-16* mutants overexpressing *dSVIP* in the gut vs. *daf-16* mutants and possible targets of HLH-30 and DAF-16. **(G)** Venn diagram illustrating common genes upregulated in both wild-type and *daf-16* mutants with *dSVIP* overexpressed in the gut. **(H)** Wikipathway enrichment analysis based on genes that were upregulated in both wild-type and *daf-16* strains overexpressing *dSVIP* in the gut.

To obtain a more holistic view of how SVIP triggers pro-health changes at the systemic level, we examined global gene expression changes caused by overexpressing *dSVIP* in the gut. We use mRNA-Seq to compare the transcriptomic profiles of wild-type animals with and without *dSVIP* gut overexpression. Surprisingly, though SVIP is not a transcription factor, we found an enormous number of differentially expressed genes upon its overexpression in the gut, including 1376 upregulated genes (Fig. 6A, Supplementary Table 3), suggesting that SVIP-dependent TL induction triggers robust metabolic shifts. We examined the identity of differentially expressed genes and, among other pathways, detected enrichment for the endoplasmic reticulum unfolded response (UPRER) (Fig. 6B), which has been demonstrated to promote longevity (Imanikia et al, 2019; Taylor & Dillin, 2013) and might contribute to the observed late age-health improvements in *SVIP*-overexpressing animals. Next, we asked whether this transcriptomic signature is reflective of HLH-30 and DAF-16 gene targets since our evidence indicates that SVIP may act on both transcription factors to trigger TL induction. To determine this, we compared the set of 1376 upregulated genes versus 1000 potential HLH-30 and DAF-16 target genes (Zou et al, 2022). We found that among the set of 1376 upregulated genes, 19 were targets of HLH-30 and 33 were targets of DAF-16 (Fig. 6C); in fact, 14 upregulated genes are predicted to be targets of both DAF-16 and HLH-30 (Fig. 6C). This finding further supports our model that SVIP acts via both transcription factors to regulate key health-promoting genes.

Because we found that SVIP bypasses DAF-16 requirements to induce TLs and promote healthy aging, we explored whether this is associated with a genetic alteration that places greater emphasis on the activation of HLH-30 target genes in the absence of *daf-16*. We analyzed the transcriptomes of *daf-16* mutants with and without *dSVIP* gut overexpression by mRNA-seq. Remarkably, we observed an even greater overall shift in differentially expressed genes in the absence of *daf-16* (Fig. 6D, Supplementary Table 3), suggesting that *daf-16* may buffer against dramatic metabolic shifts. Specifically, 2076 genes were upregulated when *dSVIP* was overexpressed in the gut of *daf-16* mutants (Fig. 6D). A functional enrichment analysis revealed a pool of activated genes enriched in age-related functions (Fig. 6E). Thus, these genes may further support the healthspan phenotype observed when *dSVIP* is overexpressed in the gut of *daf-16* mutants. To further assess the transcriptomic re-arrangement in *daf-16* mutants overexpressing *dSVIP* in the gut, we again compared the set of 2076 upregulated genes versus 1000 possible targets of HLH-30 and DAF-16 (Zou et al., 2022) and identified 64 possible targets of HLH-30 (Fig. 6F). Notably, this is three times more than when *dSVIP* was overexpressed in wild-type animals, which is in accord with our genetic evidence that SVIP may rely more heavily on HLH-30 in the absence of DAF-16. Unexpectedly, we also observed an increase in DAF-16 target genes. Given that HLH-30 and DAF-16 share many transcriptional targets, we speculate that perhaps these target genes are activated by DAF-16 under normal conditions but can also be activated by HLH-30 when DAF-16 is absent as a compensatory mechanism.

Finally, we explored whether strains overexpressing *dSVIP* in the gut, in both wild-type and *daf-16* null mutant backgrounds, share common upregulated genes by comparing their transcriptional profiles. Indeed, we observed a significant overlap between these groups (Fig. 6G). Additionally, we performed a functional pathway analysis of these common upregulated genes (Fig. 6H). Our analysis showed an enrichment in aging-related genes as well as in the gene T23F2.2, involved in the mitochondrial unfolded protein response (UPRmt), another mechanism known to mediate longevity (Shao et al, 2016). Taken together, our sequencing analyses identified transcriptional changes induced by *dSVIP* that might contribute to the healthy aging phenotypes observed in multiple *C. elegans* strains. Overall, these data demonstrate that TL induction, even in a single tissue, promotes healthy aging systemically via the concerted action of DAF-16 and HLH-30.

## Discussion

**Figure 7.**
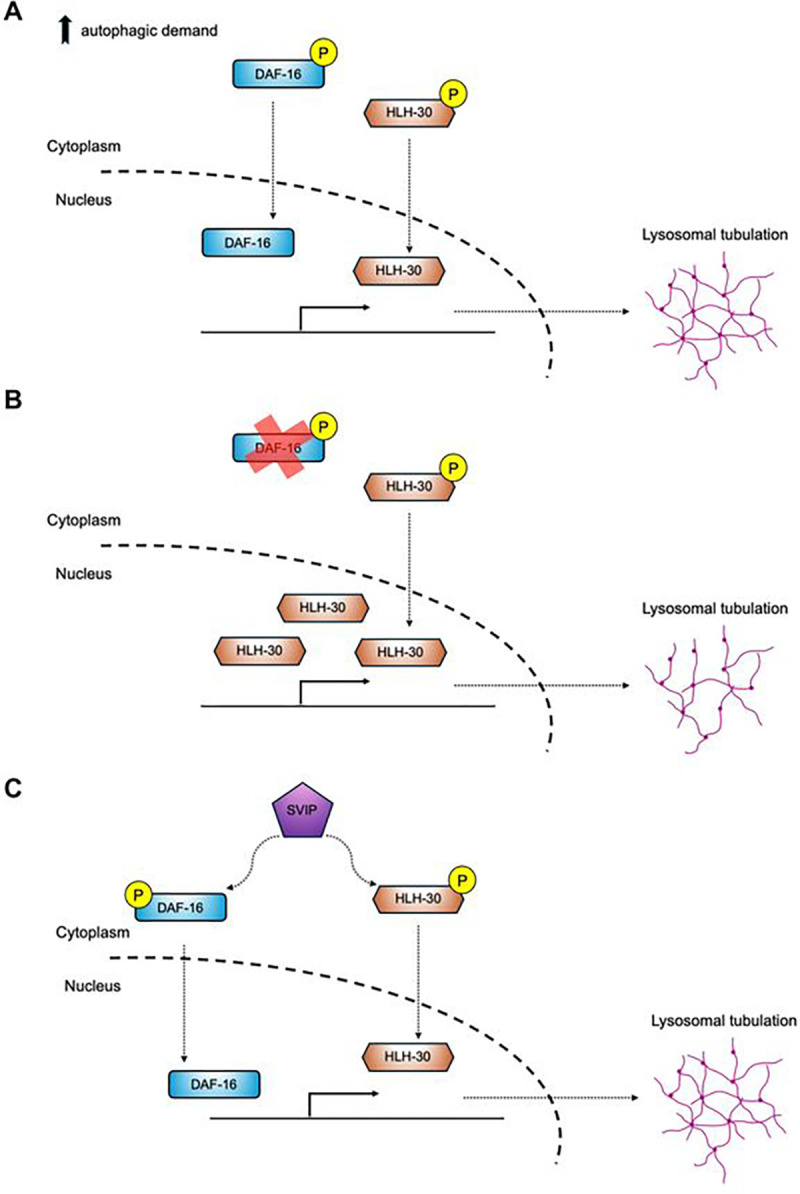
Schematic model for DAF-16 and HLH-30-dependent regulation of tubular lysosome induction. **(A)** Under high autophagic demand contexts, such as starvation and natural aging, *daf-16* and *hlh-30* cooperate to induce lysosomal tubulation in the *C. elegans* gut. **(B)** In the absence of *daf-16*, *hlh-30* overexpression compensates to induce TLs. **(C)** *SVIP* overexpression employs both *daf-16* and *hlh-30* to deploy TLs constitutively.

The global increase in life expectancy has magnified the burden of age-related diseases. DR has been an effective strategy to delay aging; however, DR implementation in the general public has many limitations. Thus, identifying strategies that can mimic the effects of DR is a major goal. In previous work, we found that constitutive induction of an atypical form of lysosomes that are tubular in structure can mimic the beneficial effects of DR and does so, in part, by amplifying cross tissue proteostasis. Thus, understanding the control mechanisms behind TL induction could inform new strategies to harness the beneficial effects of DR. In this study, we found that the collaborative action of two major pro-longevity transcription factors, DAF-16/FOXO and HLH-30/TFEB, also play a pivotal role in the formation of TLs (Fig. 7A–B). Moreover, we demonstrated that the conventional requirements to stimulate TLs in adverse conditions can be artificially bypassed through intestinal overexpression of *Drosophila SVIP*, a previously characterized TL stimulator (Fig. 7C). Finally, we observed that artificial induction of TLs via *SVIP* overexpression in the intestine caused nuclear translocation of HLH-30/TFEB across multiple tissues, leading to systemic effects that boost organismal health of aged *C. elegans*. Altogether, this work reveals a new facet of TL regulation that might be applicable in healthy aging interventions.

Although DAF-16 and HLH-30 have many distinct functions, increasing evidence suggests that, in some cases, these two transcription factors work in concert to trigger similar pro-health mechanisms, perhaps as a safeguard in the event that one transcription factor is compromised. For example, previous data indicate that DAF-16 and HLH-30 work as a transcriptional regulatory module to mediate resistance to oxidative stress (Lin et al, 2018). Furthermore, this module is required to extend lifespan through enhanced lysosomal lipolysis (Seah et al, 2016) and supports the long-lived phenotype of *daf-2* and *glp-1* mutants, as well as the regular lifespan of wild-type animals (Lin et al., 2018). Our results further indicate that the cooperation and crosstalk between the two transcription factors is required to induce certain stimuli-dependent responses; we demonstrate that DAF-16 and HLH-30 coordinate their actions to enable TL formation in contexts where there is high autophagic demand, such as during food limitation or natural aging. Mechanistically, the redundant actions of both transcription factors are likely a result of co-regulated transcriptional targets. A previous report demonstrated that DAF-16/FOXO and HLH-30/TFEB co-occupy up to 41% of target promoters and co-regulate multiple target genes (Lin et al., 2018). Consistently, our analysis of putative DAF-16 and HLH-30 direct targets from the ChIP-Atlas (Zou et al., 2022) indicate that the two TFs share up to 44% of 1000 possible target genes (Fig. 6C). These data, combined with our findings, suggest that perhaps redundancy between DAF-16/FOXO and HLH-30/TFEB is required to reinforce critical signals for health-promoting support mechanisms, such as TL induction, and if one transcription factor is lacking, the other can be stimulated to compensate and sustain TL formation (Fig. 7A–B).

A surprising finding from our study is that, although DAF-16 and HLH-30 are required to induce TLs naturally, gut *dSVIP* overexpression can still trigger TL formation in *daf-16* null mutants. How might this be occurring? Our findings suggest that in the absence of *daf-16*, SVIP relies more heavily on HLH-30 activity to bypass the normally essential requirement for DAF-16 in TL induction (Fig. 7C). Indeed, inhibition of *hlh-30* in *daf-16* mutants precluded SVIP-dependent TL induction, while experimental overexpression of *hlh-30* stimulated TL induction in *daf-16* mutants just like *SVIP* overexpression (Fig. 5B–C). Moreover, we observed a significant shift in the transcriptional program towards the activation of HLH-30-specific target genes; this could result in the expression of an alternative set of genes that could be used to deploy TLs. These observations suggest that the lysosomal machinery can be re-calibrated via different gene expression modules to regulate organismal healthspan in response to adverse conditions. Further, our data demonstrate significant crosstalk between the intestine and the muscle. Interestingly, a similar mechanism has been previously identified in *C. elegans*, in which DAF-16 initiates alternative ER-associated degradation systems to bypass the *ire-1* stress sensor required to promote ER homeostasis (Safra et al, 2014). Collectively our findings suggest that SVIP has the ability to trigger various systems to induce TLs, which confers some plasticity to efficiently support organismal health, even when one of the systems is compromised. Notably, overexpression of human *SVIP* can also stimulate TLs in the gut of well-fed *C. elegans* (Extended data, Fig. 3), suggesting that mammalian *SVIP* orthologs can also act as potent TL stimulators and provides support that the mechanisms we uncover in *C. elegans* may translate to mammalian systems.

Molecularly, how SVIP improves systemic health remains an open question. However, a major finding in our study is that constitutive induction of TLs via *dSVIP* gut overexpression results in the nuclear translocation of HLH-30 not only in the intestine but also in muscles (Fig. 5D–E). Potentially, this could explain the improved proteostasis observed across multiple tissues when TLs are deployed exclusively in the gut (Villalobos et al., 2023). We propose a model in which cell non-autonomous effects of HLH-30/TFEB mediate organismal physiology through trans-tissue signals originating in the gut. In support of our model, previous studies have demonstrated that cell non-autonomous effects of HLH-30/TFEB improve thermoresistance, proteostasis, and host defenses against *S. aureus* infections (Imanikia et al., 2019; Wani et al, 2021; Wong et al, 2023). Thus, we hypothesize that HLH-30/TFEB signals specifically emanating from the intestine are important for integrating signaling events in multiple organs. This is further supported by previous work demonstrating that intestinal signals broadcasting to muscle tissues are required to mediate stress resistance, improve systemic proteostasis, and increase longevity (Imanikia et al., 2019; Miles et al, 2023; Murphy et al, 2007; O'Brien et al, 2018; Taylor & Dillin, 2013; Zhou et al, 2019). Similarly, trans-tissue signals originating in the gut and received by the nervous system increase oxidative stress resistance and extend lifespan (Kim & Sieburth, 2018; Minnerly et al, 2017; Uno et al, 2021). Our data highlight how modulating lysosomal activity in the gut triggers HLH-30-dependent interorgan signaling events between the intestine and distal tissues to support systemic health.

If TLs are naturally stimulated during aging, why does constitutive TL stimulation by gut-specific *SVIP* overexpression further boost health in aged animals? Although we do not fully know the answer to this yet, we hypothesize that the highly digestive nature of TLs and the more efficient turnover of autophagic cargo, when TLs are present permanently from youth, provides a more robust proteostasis system to prevent cumulative tissue damage. Thus, early-life induction of TLs might help to attenuate the autophagic load at older ages by providing robust autophagic turnover throughout life. Accordingly, continuous autophagy stimulation by other approaches has been shown to extend lifespan and healthspan in various species (Carmona-Gutierrez et al, 2019; Ogasawara et al, 2020; Wang et al, 2022). Remarkably, even short-term rapamycin administration in young individuals results in prolonged autophagy activation that suppresses age-related pathologies in the gut (Juricic et al, 2022). As a corollary, it is conceivable that even brief TL induction in young animals might be sufficient to provide life-long health benefits. Notably, preventing TL induction under DR abolishes lifespan and health benefits in *C. elegans* (Villalobos et al., 2023). We envision that with increased autophagic loads, lysosomes must undergo a compensatory change in morphology to accommodate heightened turnover demands. Otherwise, lysosomes become the restrictive factor in achieving full autophagic potential. We hypothesize that the expansion of the lysosomal compartment into a tubular network increases lysosomal surface area within a cell and also potentially increases active ‘search and capture’ of molecular cargo. Our study provides new evidence of a support system that can be employed by cells to mitigate autophagic burden throughout lifespan and thereby enhance healthspan.

In summary, our study provides insights into the molecular machinery that can be used to induce robust TL formation. In theory, these mechanisms could be tapped to promote healthy aging in *C. elegans*. Our observations also indicate that early induction of TLs in the gut might further propagate pro-health signals to the whole organism to prevent age-dependent tissue deterioration. Finally, we suggest that the natural presence of TLs makes them ideal candidates to develop anti-aging interventions over other approaches, as we anticipate that their ectopic induction would have limited adverse consequences. Further studies to fine-tune their induction will be required to better exploit their activity and devise practical therapeutic strategies.

## Materials and Methods

### Strain generation

Supplementary Table 1 provides a complete list of strains used in this study. All strains used in this study were generated using standard genetic crosses or microinjection (Evans, 2006). For genetic crosses, transgenes expressing fluorescent proteins were tracked by stereomicroscopy, and gene deletions and mutations were verified by PCR and/or sequencing. For microinjection, constructs were injected individually or in combination into the gonad of adult hermaphrodites, each at a concentration of 25 ng/μl.

### Animal maintenance

Worms were raised at 20°C on NGM agar (51.3 mM NaCl, 0.25% peptone, 1.7% agar, 1 mM CaCl_2_, 1 mM MgSO_4_, 25 mM KPO_4_, 12.9 μM cholesterol, pH 6.0). Fed worms were maintained on NGM agar plates previously seeded with *E. coli* OP50 bacteria. Synchronous populations of worms were obtained by bleaching gravid hermaphrodites. Briefly, gravid worms were vortexed in 1 mL bleaching solution (0.5 M NaOH, 20% bleach) for 5 minutes to isolate eggs, and eggs were then washed three times in M9 buffer (22 mM KH_2_PO_4_, 42 mM Na_2_HPO_4_, 85.5 mM NaCl, 1 mM MgSO_4_) before plating. To obtain starved L1 animals, bleached eggs were spotted on NGM agar that lacked OP50 bacteria, and plates were maintained at 20°C for 24–48 hours before imaging. For aging experiments, synchronous populations of animals were established by bleaching gravid worms. In all aging experiments, adult worms were picked onto fresh OP50-seeded NGM plates every day to separate adults from their progeny.

### RNAi experiments

The *hlh-30* RNAi clone was obtained from the Julie Ahringer RNAi collection (Kamath & Ahringer, 2003) and verified by DNA sequencing. For RNAi experiments, synchronous populations of animals were grown on OP50-seeded NGM plates until late L4 stage, at which time they were transferred to RNAi plates (NGM plus 100 ng/μl carbenicillin and 1 mM IPTG) that had been seeded with bacteria expressing the RNAi clone. An empty L4440 vector was used as a negative control.

### VCP inhibitor treatment

A 10 μM stock solution of the VCP inhibitor CB5083 (MedChem Express, Cat. # HY-12861/CS-5405) was prepared in DMSO and diluted to a final working concentration of 1 μM in M9 buffer. 300 μl of the working stock was directly spotted onto NGM plates that were previously seeded with OP50 bacteria. For control plates, DMSO was diluted 1:10 in M9 buffer and 300 μl was directly spotted onto NGM plates that were previously seeded with OP50 bacteria. Late L4s were transferred to control (DMSO) or CB5083 plates.

### Lifespan analysis

Synchronous populations of worms were transferred as late L4s to NGM plates seeded with OP50 bacteria. Animals that exploded, bagged, or crawled off plates were censored during analysis. Lifespans were analyzed using OASIS 2 software (Han et al, 2016), and statistical significance was assessed using a log-rank test.

### Thrashing assay

Synchronous populations of animals were transferred as late L4s to NGM plates seeded with OP50 bacteria. Worms were transferred to fresh plates every day to separate adults from their progeny. To score thrashing rates, individual worms were transferred into a drop of M9 buffer on an NGM plate, and the number of body thrashes were counted in a 1-min period.

### Microscopy

For *C. elegans* whole animal imaging, 4% agarose (Fisher Bioreagents) pads were dried on a Kimwipe (Kimtech) and then placed on top of a Gold Seal^™^ glass microscope slide (ThermoFisher Scientific). A small volume of 10 mM levamisole (Acros Organics) was spotted on the agarose pad. Worms were transferred to the levamisole spot, and a glass cover slip (Fisher Scientific) was placed on top to complete the mounting. To determine HLH-30::GFP localization worms were analyzed within 3 minutes once mounting was completed.

### Image analysis

Images were processed using LAS X software (Leica) and FIJI/ImageJ (NIH). Lysosome networks were analyzed using “Skeleton” analysis plugins in FIJI. Briefly, images were converted to binary 8-bit images and then to skeleton images using the “Skeletonize” plugin. Skeleton images were then quantified using the “Analyze Skeleton” plugin. Number of objects, number of junctions, and object lengths were scored. An “object” is defined by the Analyze Skeleton plugin as a branch connecting two endpoints, an endpoint and junction, or two junctions. Junctions/object was used as a parameter to quantify network integrity.

For analyzing fluorescence intensity, the gut tissue was outlined using the free-draw tool in FIJI/ImageJ, and average fluorescence intensity of the outlined area was measured. For all intensity experiments, 50% laser intensity, 300 ms exposure time, and 100% Fluorescence Intensity Manager settings were used.

### Statistical analyses

Data were statistically analyzed using GraphPad Prism. For two sample comparisons, an unpaired t-test was used to determine significance (a=0.05). For three or more samples, a one-way ANOVA with Dunnett’s, Tukey’s, or Šídák’s multiple comparisons was used to determine significance (a=0.05). For grouped comparisons, a two-way ANOVA with Šídák’s multiple comparisons was used to determine significance (a=0.05). Statistical significance of lifespan data was determined using a log-rank test.

### RNA Sequencing

Gravid adult worms were bleached, and eggs were plated onto NGM plates to produce synchronized populations of worms. For each genotype, day 1 adult worms were collected in M9 in three independent biological replicates. RNA extraction was done using standard a TRIzol TM reagent protocol (Thermo Fisher Scientific, cat# 15596018). Subsequently, genomic DNA removal was performed using a GeneJet RNA-purification kit (Thermo Fisher Scientific, cat# K0702). The concentration of purified RNA was measured using a nanodrop and quality was assessed using a Bioanalyzer. At least 400ng/μl of Purified RNA for each replicate was sent to Novogene for cDNA library preparation and Illumina sequencing (Illumina NovaSeq 6000).

Sequencing reads were mapped to the *C. elegans* reference genome (WBcel235) using HISAT2 (Pertea et al, 2016). We used featureCounts v1.5.0-p3 (Liao et al, 2014) to count the reads mapped to each gene and calculate FPKM. We also used Salmon (Patro et al, 2017) to quantify gene expression in alignment-based mode. Differential expression analyses was performed using the DESeq2 R package (1.20.0) (Love et al, 2014). DESeq2 provides statistical routines for determining differential expression in digital gene expression data using a model based on the negative binomial distribution. The resulting p-values were adjusted using the Benjamini and Hochberg’s approach for controlling the false discovery rate (FDR). We used adjusted p-value ≤ 0.05 and fold-change ≥ 2 as a cut-off for differentially expressed genes. Differentially expressed genes were analyzed with enrichR (Kuleshov et al, 2016) to look for enriched gene sets (adjusted p-value ≤ 0.05) with respect to WikiPathways database (Agrawal et al, 2023).

## Data Availability

All data are available in the main text or the supplementary materials. Additional information on data sources is available upon request from the corresponding author. All unique materials used in the study are available from the authors or from commercially available sources. For the gene expression analyses, the raw and processed data have been submitted to NCBI under the BioProject accession PRJNA1083209. We used the same bioinformatics pipeline used in Pandey et al (2023) (Pandey et al, 2023), which is available at github at https://github.com/pkerrwall/dec2_fly.
